# Natural environmental factors at birth on risk for rheumatoid arthritis: the impact of season, temperature, latitude, and sunlight exposure

**DOI:** 10.1186/s12889-025-22448-2

**Published:** 2025-04-03

**Authors:** Liang Luo, Jia Yi Hee, Sitian Zang, Zhike Liu, Kun Tang, Xuewu Zhang, Chun Li

**Affiliations:** 1 Department of Chinese Medicine, The People’s Hospital of Yubei District of Chongqing, No. 23, North Central Park Road, Yubei District, Chongqing, China; 2 Department of Rheumatology and Immunology, Peking University People’s Hospital, No. 11 Xizhimen South Street, Xicheng District, Beijing, China; 3 Vanke School of Public Health, Tsinghua University, Zhongguancun North Street, Haidian District, Beijing, China; 4 Department of Epidemiology and Biostatistics, Peking University Health Science Center, No. 38 Xue Yuan Road, Haidian District, Beijing, China

**Keywords:** Rheumatoid arthritis, Risk factor, Exposure, Environment

## Abstract

**Background:**

Environmental factors contribute to approximately 41% of the risk of rheumatoid arthritis (RA). Previous studies have focused on anthropogenic environmental factors, while much less attention has been given to natural environmental factors. Our study explored the potential influence of natural environmental factors at birth on the risk of RA.

**Methods:**

This large retrospective study utilized data from the China Kadoorie Biobank. A restricted cubic spline (RCS) model was employed to explore nonlinear relationships between natural environmental factors and the risk of RA. Additionally, a multivariable Cox regression model, adjusted for confounding factors, was used to examine correlations between season of birth, geographic, climate, and the risk of RA.

**Results:**

A total of 512,715 participants were included in this study, of which 2889 (0.56%) were diagnosed with RA. The RCS analysis revealed that the monthly average temperature at birth (*p* < 0.001), the latitude (*p* = 0.027) of the birthplace, and the sunshine rate (*p* < 0.001) exhibited a nonlinear relationship with the risk of RA. Multivariable Cox regression analysis revealed that participants born in Spring and Summer (HR 1.13, 95% CI 1.05–1.23) had an increased risk of RA compared to those born in Autumn and Winter. Additionally, participants born at latitudes ≤ 24°N (HR 1.49, 95% CI 1.32–1.68), with sunshine rate ≤ 28% (HR 2.00, 95% CI 1.75–2.29) or ≥ 60% (HR 1.22, 95% CI 1.08–1.38) had an increased risk of RA. Being born in regions with a monthly average temperature ≥ 27 °C (HR 0.82, 95% CI 0.72–0.95) was associated with a decreased risk of RA.

**Conclusion:**

Being born in Spring and Summer, as well as early-life exposure to low-latitude regions and extreme sunlight environments increases the risk of RA. Our study revealed significant associations between the risk of RA and natural environmental factors at birth, emphasizing the impact of the early-life environment on the onset of RA.

**Supplementary Information:**

The online version contains supplementary material available at 10.1186/s12889-025-22448-2.

## Introduction

It has been estimated that genetic factors such as human leukocyte antigen alleles account for up to 60% of rheumatoid arthritis (RA) risk factors, while environmental factors (i.e., describing susceptibility factors unrelated to genetics including for example diet, air-borne exposures, infections) account for the remaining risk of RA [1, 2]. Typically, RA affects approximately 2% of the population, although these proportions vary from 0.5% in Oceania to 4.3% in Andean Latin America [3]. The incidence of RA appears to be on the rise at the global level, suggesting that environmental and lifestyle factors influence RA.

The association between the season at birth and musculoskeletal diseases was first put forward by Fønnebø in 1987 [4]. For the period from 1970 to 1986, Fønnebø reported a significantly higher incidence of musculoskeletal disorders (myalgia, peritendinitis, lumbago, and osteoarthritis) in patients presenting to a rehabilitation centre with birth months of June and July, and noted the lowest incidence of musculoskeletal disorders in patients presenting to the rehabilitation centre with the birth month of December [4]. The number of patients with musculoskeletal disorders was twice as high for patients born in Summer months as for patients born in Winter months [4]. Positive associations between the month or season at birth have also been reported with dementia [5], diabetes [6], and multiple sclerosis [7]. A study of patients with seropositive erosive RA performed by Buchanan et al. found no difference between the incidence of RA and birth season [8].

According to the hypothesis on the fetal origin of adult disease, seasonal factors can influence the risk of physiological and pathological conditions in adulthood, owing to environmental influences on embryonic or foetal structures [9, 10]. This suggests that the time of the year at birth may influence susceptibility to disease in later life as seasonal variations can result in varying factors such as levels of sunlight, nutrition, and air pollution [11, 12]. While extensive research recognized environmental contributors such as smoking, diet, and air pollution [13], relatively few studies have examined how early-life exposures, including seasonal environmental variations at birth, might predispose individuals to RA later in life. Seasonality affects several factors known to be associated with the risk of RA [14–16]. For example vitamin D levels, nutrition, temperature, humidity, and levels of particulate matter. By integrating epidemiological data with environmental exposure metrics, our study aims to investigate potential associations between natural environmental factors at birth and the risk of RA.

## Methods

### Data sources and inclusion criteria

This study used data from the China Kadoorie Biobank (CKB), a prospective study initiated by the University of Oxford and the Chinese Centre for Disease Control and Prevention. The CKB assessed and followed up a total of 0.5 million Chinese individuals to collect determinants of a wide range of common diseases. The study design and methods of the CKB database have been described in detail elsewhere [17, 18]. The historical monthly average surface temperature and monthly average relative humidity data were provided by the China Meteorological Administration.

Inclusion criteria for the CKB database included eligible participants selected for the study within each of 10 regions (see below) through official residential records, selected participants in possession of a unique national identity card, and selected participants aged between 30 and 79 years. All participants provided written informed consent according to the Declaration of Helsinki for participation and to allow for access to their medical records [17]. The CKB database has been given ethics approval by the University of Oxford, the Chinese Centre for Disease Control and Prevention (CDC), and the institutional research boards of the local CDCs in the study regions. All participants were included in the final analyses.

Between 2004 and 2008, a total of 302,510 women and 210,205 men were recruited from five urban (Qingdao, Harbin, Haikou, Suzhou, and Liuzhou) and five rural (Chengdu, Tianshui, Huixian, Tongxiang, and Liuyang) areas of China. The areas were chosen according to local disease patterns, exposure to risk factors of interest, population stability, quality of local disease and death registries, and local commitment and capacity to carry out the study prospectively [17]. Characteristics and locations of included regions are shown in Supplementary Tables 1 and supplementary Fig. 1.

### Procedures and bias mitigation

At local community assessment centres in each study area, trained medical staff with research experience administered an electronic questionnaire that included, but was not limited to, questions on sociodemographic status, lifestyle habits, and medical history [17]. Measurements such as height and weight were also collected by trained technicians according to standard protocols. Repeated sampling of selected items in the questionnaire and measurements was carried out randomly in approximately 3% of participants from each community to ensure data consistency and quality.

### Variables and definitions

The outcome of interest for this study was the diagnosis of RA. All patients with RA met the 1987 classification criteria for RA [19], and were confirmed by physicians. The main variables of interest were natural environmental factors at birth, including month, season, surface temperature, relative humidity, latitude, and sunshine rate (Supplementary Table 1). Other variables of interest included, but not limited to, sex, age, region (urban or rural, coastal, mountain, and plains), chronic diseases (cardiovascular disease, diabetes, chronic obstructive pulmonary disease, and asthma), alcohol consumption, and tobacco use. Climate was categorized as tropical/subtropical or temperate.

### Statistical analysis

Categorical variables were described as proportions (percentage), and continuous variables were expressed as means ± standard deviation for variables with normal distribution, or as medians (interquartile range) for variables with skewed distribution (assessed using the Kolmogorov-Smirnov test). Categorical variables were compared using the chi-square test. Continuous variables were compared using one-way analysis of variance for variables with normal distribution and the Kruskal-Wallis test for variables with skewed distribution. There were no missing values.

Based on the extensive report on RA incidence (0.28%) in Beijing, China [20], we computed the expected number of RA cases for each month (Supplementary Table 2) to account for temporal fluctuations in general population births and isolate seasonal patterns potentially linked to RA risk. Observed-to-expected birth ratios of RA were calculated to identify deviations from baseline birth distributions, which may reflect early-life environmental exposures. Logistic regression was performed to obtain the odds ratio (OR) and 95% confidence intervals (CI) for the association between month at birth and the risk of RA.

Restricted cubic splines (RCS) with three knots at the 10th, 50th, and 90th percentiles were applied to investigate the nonlinear correlation between natural environmental factors at birth and the risk of RA. Variables (temperature, latitude, and sunshine rate) showing a non-linear relationship with the risk of RA were classified into three categories according to the RCS cutoff points. The natural environmental variables with *p* < 0.05 in the univariate Cox regression were entered into a multivariate Cox regression model to estimate their associations with risk of RA, reported as hazard ratios (HR) and 95%CI. The models were adjusted for sex, alcohol consumption, tobacco use, breast-feeding, chronic diseases, and geographical region (coastal, mountain, and plains). Considering the significant difference in RA incidence between males and females, we fitted models for each sex separately.

Differences were considered statistically significant at *p* < 0.05. All statistical models were performed using SPSS version 26.0 (SPSS, Chicago, IL, USA) and R Studio version 1.3 (R Studio, Boston, MA).

## Results

The characteristics of the participants stratified by RA status are presented in Table 1. Among the participants, 0.56% had RA with a median age at RA diagnosis of 45.0 (35.0, 54.0) years. Natural environmental factors at birth, including region (*p* < 0.001), temperature (15.2 ± 10.1 °C vs. 14.8 ± 10.5 °C, *p* = 0.033), humidity (75.5 ± 10.0% vs. 74.5 ± 10.7%, *p* < 0.001), latitude (31.1 ± 6.5 °N vs. 31.8 ± 6.2 °N, *p* < 0.001), and sunshine rate (43.9 ± 10.5% vs. 45.0 ± 9.9%, *p* < 0.001), also exhibited significant differences.

**Table 1:** Comparison of characteristics between RA patients and the general population

	Total (*n* = 512,715)	RA patients (*n* = 2889)	General population (*n* = 509,826)	*p*-value
Sex				< 0.001
Male	210,205 (41.0)	793 (27.4)	209,412 (41.1)	
Female	302,510 (59.0)	2096 (72.6)	300,414 (58.9)	
Age at study, years	51.5 (42.9, 59.7)	56.3 (49.9, 64.6)	51.5 (42.9, 59.6)	< 0.001
Region				0.808
Urban	226,182 (44.1)	1268 (43.9)	224,914 (44.1)	
Rural	286,533 (55.9)	1621 (56.1)	284,912 (55.9)	
Climate				0.032
Tropical/Subtropical	306,408 (59.8)	1783 (61.7)	304,625 (59.8)	
Temperate	206,307 (40.2)	1106 (38.3)	205,201 (40.2)	
Smoking				< 0.001
Non-smoker	317,486 (61.9)	2049 (70.9)	315,437 (61.9)	
Smoker	195,229 (38.1)	840 (29.1)	194,389 (38.1)	
Alcohol consumption				< 0.001
Non-alcohol drinker	235,103 (45.9)	1563 (56.1)	233,540 (45.8)	
Alcohol drinker	277,612 (54.1)	1326 (45.9)	276,286 (54.2)	
Natural environmental factors at birth				
Season				0.053
Spring/Summer	242,109 (47.2)	1716 (0.6)	240,693 (99.4)	
Autumn/Winter	270,606 (52.8)	1473 (0.5)	269,133 (99.5)	
Month				0.478
January	40,986	214 (0.5)	40,772 (99.5)	
February	40,781	208 (0.5)	40,573 (99.5)	
March	40,753	245 (0.6)	40,508 (99.4)	
April	38,325	238 (0.6)	38,087 (99.4)	
May	38,635	230 (0.6)	38,405 (99.4)	
June	37,563	211 (0.6)	37,352 (99.4)	
July	41,514	225 (0.5)	41,289 (99.5)	
August	45,319	267 (0.6)	45,052 (99.4)	
September	45,003	234 (0.5)	44,769 (99.5)	
October	51,241	303 (0.6)	50,938 (99.4)	
November	46,412	260 (0.6)	46,152 (99.4)	
December	46,183	254 (0.6)	45,929 (99.5)	
Region				< 0.001
Chengdu	55,686	504 (0.9)	55,182 (99.1)	
Liuyang	59,900	262 (0.4)	59,638 (99.6)	
Liuzhou	50,173	339 (0.7)	49,834 (99.3)	
Tongxiang	57,704	178 (0.3)	57,526 (99.7)	
Suzhou	53,259	228 (0.4)	53,031 (99.6)	
Haerbin	57,556	303 (0.5)	57,253 (99.5)	
Huixian	63,356	241 (0.4)	63,115 (99.6)	
Qingdao	35,508	126 (0.4)	35,382 (99.6)	
Tianshui	49,887	436 (0.9)	49,451 (99.1)	
Haikou	29,686	272 (0.9)	29,414 (99.1)	
Temperature, °C	14.8 ± 10.5	15.2 ± 10.1	14.8 ± 10.5	0.033
Humidity, %	74.5 ± 11.0	75.5 ± 10.0	74.5 ± 10.7	< 0.001
Latitude, °N	31.8 ± 6.2	31.1 ± 6.5	31.8 ± 6.2	< 0.001
Sunshine rate, %	45.5 ± 9.9	43.9 ± 10.5	45.0 ± 9.9	< 0.001

Data are medians (IQR), n (%), or means (SD). RA = rheumatoid arthritis; IQR = interquartile range

**Fig. 1:**
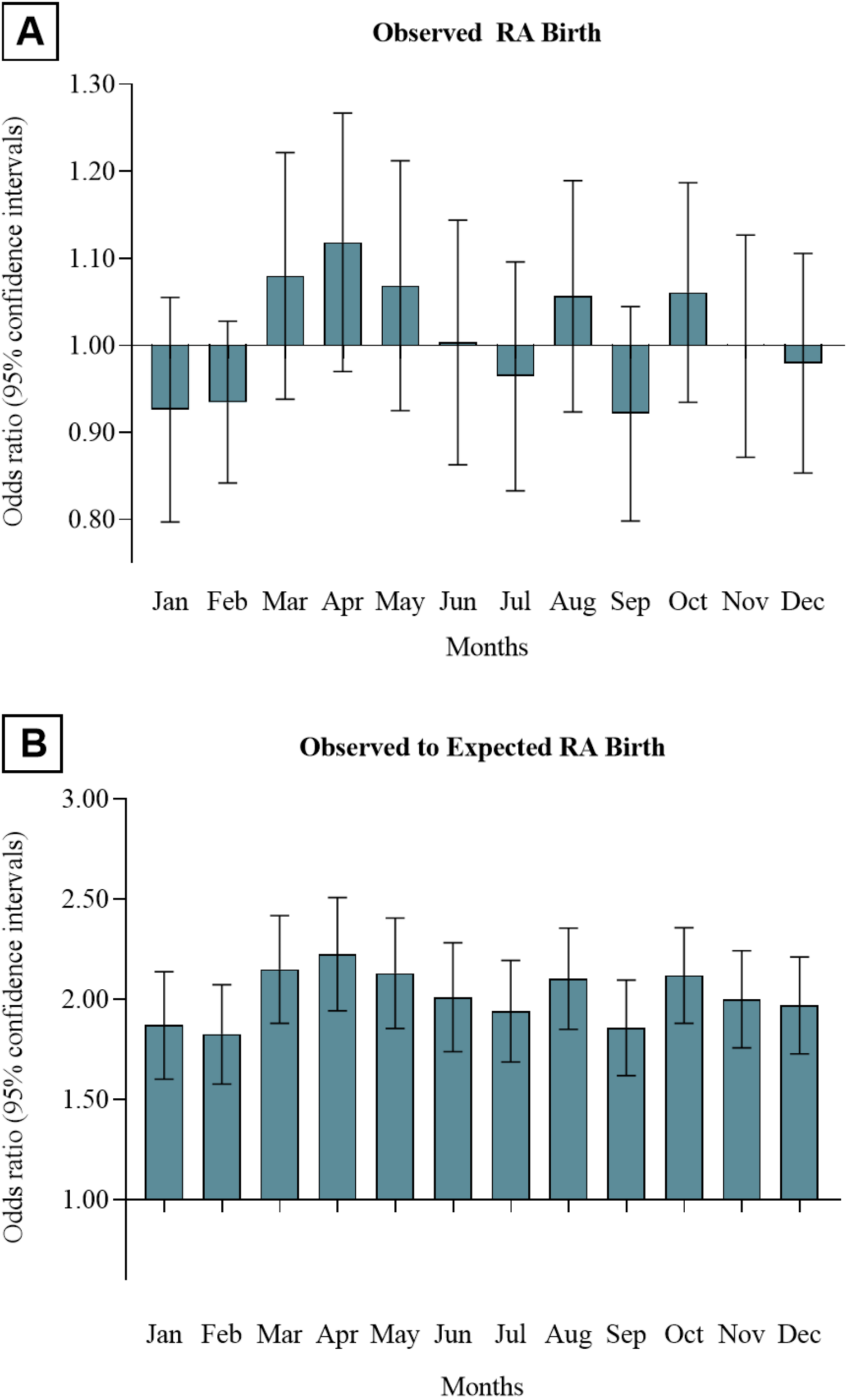
Residual differences in RA births per month. **A**, Observed RA birth ratios in the CKB cohort. **B**, Observed to expected RA birth ratios in the CKB cohort. Error bars indicate the 95% confidence intervals

Further analyses indicate that there was no significant difference in RA incidence across different months within each region. There was no significant association between the month of birth and the risk of RA (Fig. 1A). Upon further analysis, we observed that the peak-trough months were April (observed to expected birth ratio, OR 2.22, 95% CI 1.94–2.51), with 0.34% more RA births, and February (observed to expected birth ratio, OR 1.83, 95% CI 1.58–2.07), with 0.23% more RA births than expected (Fig. 1B).

**Fig. 2:**
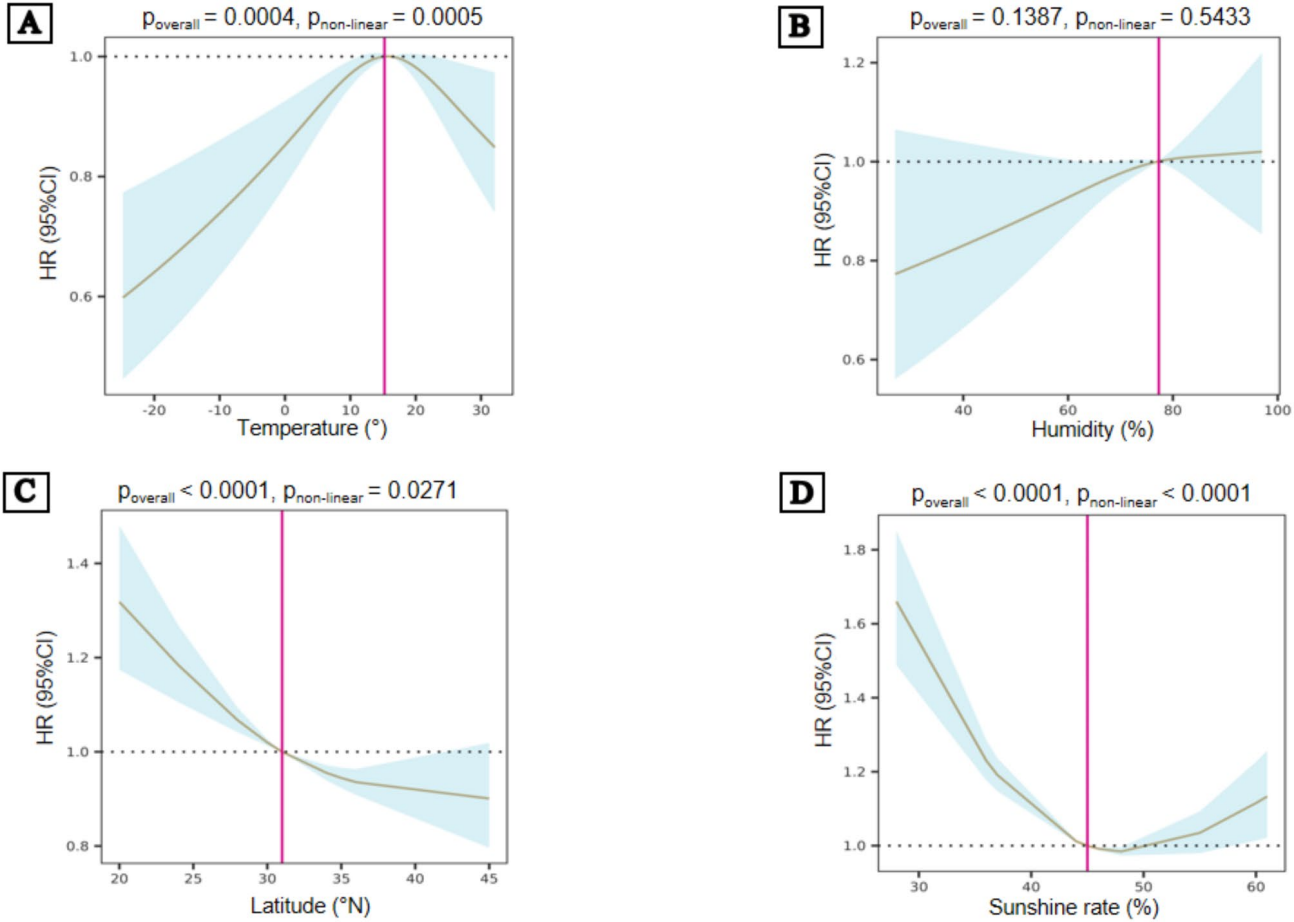
Association between natural environment factors and the risk of RA. The non-linear association between temperature (**A**), latitude (**C**), and sunshine rate (**D**) at birth with the risk of RA, fitted with a restricted cubic spline in COX regression models. The linear association between humidity (**B**) and the risk of RA using restricted cubic splines. HR = hazard ratio; CI = confidence intervals

In the study population, the monthly average temperature at birth (*p* < 0.001), the latitude (*p* = 0.027) of the birthplace, and the sunshine rate (*p* < 0.001) demonstrated a nonlinear relationship with the risk of RA, whereas the monthly average humidity (*p* = 0.543) at birth was linearly associated with the risk of RA (Fig. 2).

After adjusting for tobacco use, alcohol consumption, sex, breastfeeding, chronic diseases and geographical region, a multivariable Cox regression analysis revealed that participants born in Spring and Summer (HR 1.13, 95% CI 1.05–1.23) had a higher risk of RA compared to those born in Autumn and Winter. Additionally, participants born at latitude ≤ 24 °N (HR 1.49, 95% CI 1.32–1.68), with sunshine rate ≤ 28% (HR 2.00, 95% CI 1.75–2.29) or ≥ 60% (HR 1.22, 95% CI 1.08–1.38) had a higher risk of RA. Being born with a monthly average temperature ≥ 27 °C (HR 0.82, 95% CI 0.72–0.95) was associated with a decreased risk of RA (Table 2).

**Table 2:** The association between natural environmental factors at birth and the risk of RA in the entire population

Variables	Univariate			Multivariate	
	*N* (%)	HR (95%CI)	*p*-value	HR (95%CI)	*p*-value
Season					
Spring/Summer	242,109 (47.2)	1.10 (1.02–1.18)	0.014	1.13 (1.05–1.23)	0.002
Autumn/Winter	270,606 (52.8)	Reference	N/A	Reference	N/A
Temperature, °C					
≤ 2.3	52,482 (10.2)	0.85 (0.75–0.97)	0.012	1.06 (0.92–1.23)	0.421
2.3–27	407,547 (79.5)	Reference	N/A	Reference	N/A
≥ 27	52,686 (10.3)	0.85 (0.75–0.96)	0.012	0.82 (0.72–0.95)	0.006
Humidity, %	512,715 (100)	1.00 (1.00-1.01)	0.049	1.00 (0.99-1.00)	0.401
Latitude, °N					
≤ 24	79,859 (15.6)	1.28 (1.16–1.40)	< 0.001	1.49 (1.32–1.68)	< 0.001
24–45	375,300 (73.2)	Reference	N/A	Reference	N/A
≥ 45	57,556 (11.2)	0.89 (0.79-1.00)	0.057	0.98 (0.84–1.15)	0.835
Sunshine rate, %					
≤ 28	55,686 (10.9)	1.91 (1.71–2.13)	< 0.001	2.00 (1.75–2.29)	< 0.001
28–60	163,332 (31.9)	Reference	N/A	Reference	N/A
≥ 60	293,697 (57.2)	1.10 (1.01–1.19)	0.031	1.22 (1.08–1.38)	0.002
Alcohol	277,612 (54.1)	0.80 (0.74–0.85)	< 0.001	1.00 (0.92–1.09)	0.958
Smoking	194,993 (38.0)	0.61 (0.56–0.66)	< 0.001	0.98 (0.86–1.10)	0.688
Female	302,510 (59.0)	2.00 (1.84–2.17)	< 0.001	1.93 (1.30–2.86)	0.001
Breast-feeding	298,406 (58.2)	1.97 (1.81–2.13)	< 0.001	1.00 (0.69–1.47)	0.982
Geographical region					
Coastal	65,194 (12.7)	0.97 (0.83–1.09)	0.631	0.85 (0.74–0.98)	0.028
Mountain	273,350 (53.3)	Reference	N/A	Reference	N/A
Plains	174,171 (34.0)	0.70 (0.64–0.76)	< 0.001	0.81 (0.72–0.91)	< 0.001
Chronic diseases	65,407 (12.8)	1.12 (1.02–1.23)	0.014	1.13 (1.03–1.24)	0.009

Data are n (%). N/A = not applicable. Adjusted for sex, alcohol consumption, tobacco use, breast-feeding, geographical region, and chronic diseases. Chronic diseases include cardiovascular disease, diabetes, chronic obstructive pulmonary disease, and asthma

**Fig. 3:**
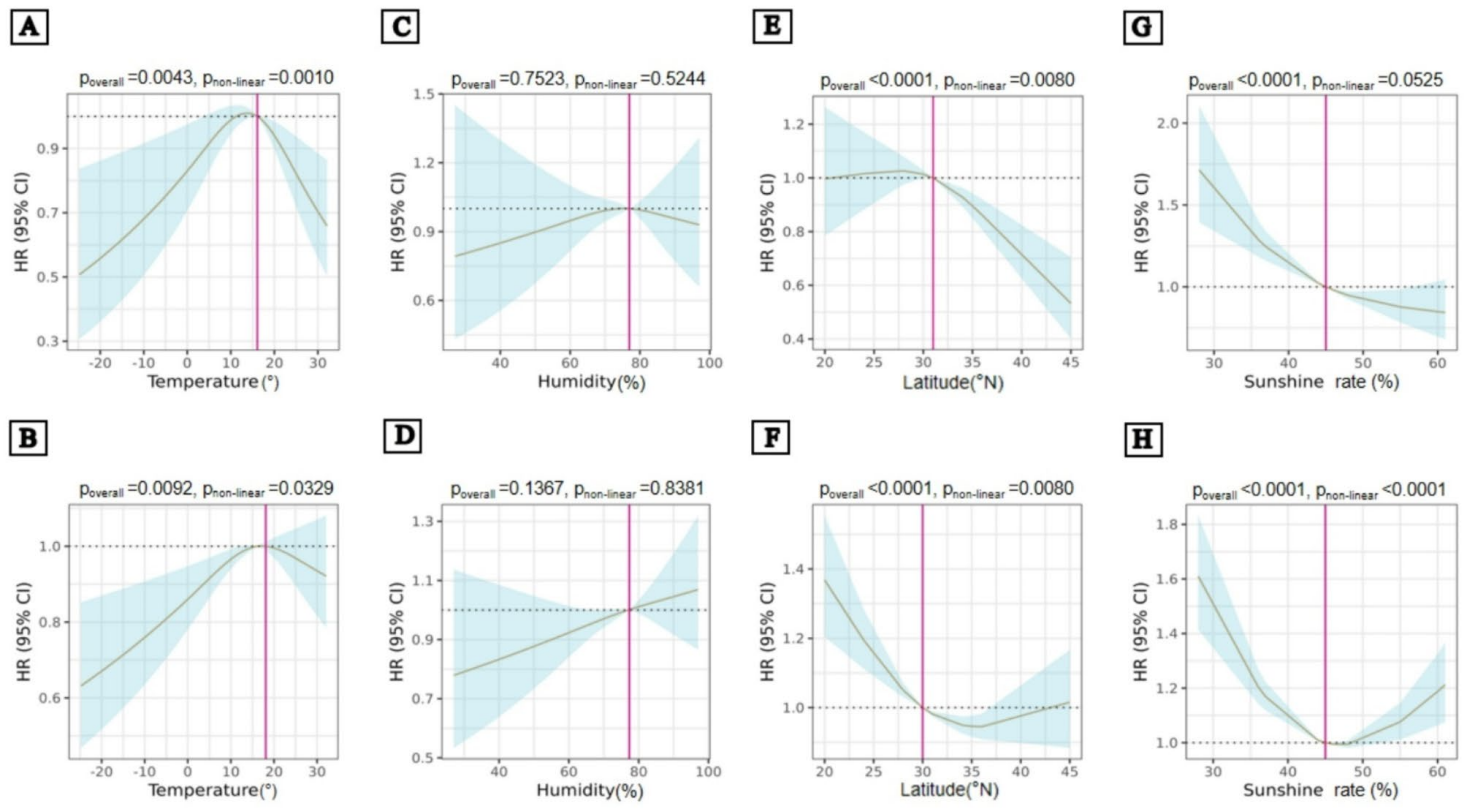
Association between natural environment factors and the risk of RA in each sex. The non-linear association between temperature (**A**, **B**), and latitude (**E**, **F**) at birth with the risk of RA in males and females, fitted with a restricted cubic spline in COX regression models. A linear relationship exists between sunshine rate and the risk of RA in males (**G**), compared to the non-linear relationship observed in females (**H**). The linear association between humidity and the risk of RA in males (**C**) and females (**D**) using restricted cubic splines

The non-linearity tests for monthly average temperature, monthly average humidity, and latitude at birth, were consistent across participants of different sexes and the entire population. Males showed a linear relationship between sunshine rate and the risk of RA, while females showed a non-linear relationship (Fig. 3).

After adjusting for tobacco use, alcohol consumption, chronic diseases and geographical region, a multivariable Cox regression analysis revealed that in males, the risk of RA decreased when the monthly average temperature at birth was ≥ 27 °C (HR 0.70, 95% CI 0.53–0.92) or the latitude at birth was ≥ 45°N (HR 0.58, 95% CI 0.41–0.82; Table 3). When exploring the relationship between natural environmental factors and the risk of RA in female participants, adjustments were made for tobacco use, alcohol consumption, breastfeeding, chronic diseases and geographical region. A multivariable Cox regression analysis revealed that females born in Spring and Summer (HR 1.15, 95% CI 1.04–1.26; Table 4) had an increased risk of RA compared to those born in Autumn and Winter. Additionally, consistent with the entire population, both male and female subgroup analyses demonstrated an increased risk of RA when latitude was ≤ 24°N or when the sunshine rate was either ≤ 28% or ≥ 60% (Tables 3 and 4).

**Table 3:** Natural environmental factors for the risk of RA in males

Variables	Univariate			Multivariate	
	*N* (%)	HR (95%CI)	*p*-value	HR (95%CI)	*p*-value
Season					
Spring/Summer	98,158 (46.7)	0.98 (0.86–1.13)	0.825		
Autumn/Winter	112,047 (53.3)	Reference	N/A		
Temperature, °C					
≤ 2.1	21,051 (10.0)	0.87 (0.68–1.11)	0.264	1.308 (1.00-1.71)	0.050
2.1–27	167,733 (79.8)	Reference	N/A	Reference	N/A
≥ 27	21,421 (10.2)	0.70 (0.54–0.91)	0.009	0.70 (0.53–0.92)	0.011
Humidity, %	210,205 (100)	1.00 (1.00-1.01)	0.665		
Latitude, °N					
≤ 24	30,115 (14.3)	1.07 (0.90–1.29)	0.465	1.36 (1.08–1.71)	0.009
24–45	156,838 (74.6)	Reference	N/A	Reference	N/A
≥ 45	23,252 (11.1)	0.51 (0.39–0.68)	< 0.001	0.58 (0.41–0.82)	0.002
Sunshine rate, %					
≤ 28	21,315 (10.1)	2.05 (1.66–2.52)	< 0.001	1.99 (1.57–2.53)	< 0.001
28–60	68,054 (32.4)	Reference	N/A	Reference	N/A
≥ 60	120,836 (57.5)	1.07 (0.91–1.25)	0.434	1.29 (1.05–1.57)	0.013
Alcohol	167,425 (79.6)	0.94 (0.80–1.11)	0.465	0.96 (0.82–1.14)	0.663
Smoking	179,899 (85.6)	1.13 (0.92–1.37)	0.242	1.09 (0.89–1.34)	0.387
Geographical region					
Coastal	26,418 (12.6)	0.81 (0.65–1.01)	0.057	0.70 (0.54–0.92)	0.011
Mountain	110,331 (52.5)	Reference	N/A	Reference	N/A
Plains	73,456 (34.9)	0.55 (0.47–0.65)	< 0.001	0.70 (0.57–0.86)	0.001
Chronic diseases	28,972 (13.8)	0.94 (0.78–1.13)	0.492	0.94 (0.78–1.12)	0.474

Data are n (%). Adjusted for alcohol consumption, tobacco use, geographical region, and chronic diseases

**Table 4:** Natural environmental factors for the risk of RA in females

Variables	Univariate			Multivariate	
	*N* (%)	HR (95%CI)	*p*-value	HR (95%CI)	*p*-value
Season					
Spring/Summer	143,951 (47.6)	1.13 (1.04–1.23)	0.005	1.15 (1.04–1.26)	0.004
Autumn/Winter	158,559 (52.4)	Reference	N/A	Reference	N/A
Temperature, °C					
≤ 2.3	30,525 (10.1)	0.84 (0.72–0.97)	0.020	0.97 (0.82–1.16)	0.763
2.1–27	240,720 (79.6)	Reference	N/A	Reference	N/A
≥ 27	31,265 (10.3)	0.90 (0.78–1.04)	0.143	0.89 (0.76–1.04)	0.136
Humidity, %	302,510 (100)	1.00 (1.00-1.01)	0.041	1.00 (1.00-1.01)	0.930
Latitude, °N					
≤ 24	49,744 (16.4)	1.31 (1.18–1.45)	< 0.001	1.56 (1.35–1.79)	< 0.001
24–45	218,462 (72.1)	Reference	N/A	Reference	N/A
≥ 45	34,304 (11.3)	1.02 (0.89–1.17)	0.778	1.15 (0.96–1.39)	0.128
Sunshine rate, %					
≤ 28	34,371 (11.4)	1.82 (1.59–2.07)	< 0.001	2.10 (1.78–2.47)	< 0.001
28–60	95,278 (31.5)	Reference	N/A	Reference	N/A
≥ 60	172,861 (57.1)	1.10 (1.00-1.21)	0.060	1.24 (1.07–1.43)	0.005
Alcohol	110,187 (36.4)	1.07 (0.98–1.17)	0.129	1.02 (0.93–1.12)	0.695
Smoking	15,330 (5.1)	1.12 (0.96–1.32)	0.161	0.86 (0.72–1.02)	0.079
Breast-feeding	298,368 (98.6)	1.01 (0.69–1.47)	0.968	1.02(0.70–1.49)	0.918
Geographical region					
Coastal	38,776 (12.8)	1.02 (0.90–1.15)	0.821	0.90 (0.76–1.07)	0.239
Mountain	163,019 (53.9)	Reference	N/A	Reference	N/A
Plains	100, 715 (33.3)	0.76 (0.69–0.84)	< 0.001	0.87 (0.76-1.00)	0.050
Chronic diseases	36,435 (12.0)	1.24 (1.11–1.38)	< 0.001	1.22 (1.09–1.36)	< 0.001

Data are n (%). Adjusted for alcohol consumption, tobacco use, breast-feeding, geographical region, and chronic diseases

## Discussion

In this study, we found that participants born in Spring and Summer had an increased risk of RA. In February and April, there were significant fluctuations in the birth rates of RA patients compared to the expected rates. Natural environmental factors at birth, including temperature, latitude, and sunshine exposure, were significantly associated with the risk of RA.

The interaction between genetic and environmental risk factors is considered a primary driver of the risk of RA, with environmental factors contributing to 41% of the risk of RA [13, 21, 22]. In addition, recent genetic studies highlight that key regulators of bone marrow mesenchymal stem cells, such as the miR-665/SOST axis and ERα signaling pathways [23, 24], may further modulate bone remodeling and immune-microenvironment crosstalk. Seasonal changes can lead to changes in host immune competence [25]. The research findings on the seasonality of the risk of RA are inconsistent. In contrast to our findings, in a United States RA cohort, lower odds of RA were found in patients born in Autumn month of October, but also in the Summer month of August [26], while another study reported an elevated risk of RA for patients born in Winter [27]. Other relevant studies of RA and juvenile RA did not report associations between month at birth and the risk of RA [8, 28].

Contrary to previous studies [29–31], although we did not find an overall significant association between birth month and the risk of RA, we observed a significant fluctuation in the observed-to-expected RA birth ratio within certain months. Most notably, we found a substantial increase in RA incidence rates for those born in February and April compared to expected rates. A case-control study conducted in South Korea also found that RA patients born in March reached the annual peak [32]. The considerable spikes in RA rates among births in specific months suggest environmental factors during critical prenatal or early life periods could interact with genetic factors to shape the risk of RA. There is evidence that seasonal variation at birth is associated with the development of autoimmune diseases later in life [33]. Therefore, further investigation is warranted into the potential seasonal environmental triggers underlying the detected month-to-month variability in RA.

Some studies have documented associations between temperature and RA disease activity [34–36]. However, research exploring the impact of temperature on the risk of RA remains limited. Recent research has found a positive association between exposure to cold environments and the risk of RA [37]. In our study, we identified a non-linear relationship between temperature and the risk of RA. Specifically, different temperature ranges were linked to varying levels of the risk of RA, indicating that these environmental variables may directly affect the immune pathways of autoimmunity [38].

The geographical location at birth also showed correlations with the risk of RA. Research in different regions revealed geographic variations in the risk of RA [39–42]. A worldwide cross-sectional analysis showed that patients living in low-latitude regions develop RA at a younger age, highlighting the long-term impact of early-life environmental factors on RA susceptibility [43]. Contrary to our findings, surveys conducted in the northern United States have suggested that women residing in high-latitude regions may be at a higher risk of RA [40]. The puzzling geographical variations in the risk of RA lack a clear explanation, suggesting the involvement of complex interactions between genetic factors and geographic variables.

Interestingly, our results demonstrated a positive association between excessive or insufficient sunlight exposure at birth and an increased risk of developing RA later in life after adjusting for confounding factors. One prominent hypothesis for the pathogenesis of RA implicates vitamin D deficiency resulting from inadequate sunlight exposure (ultraviolet) [40, 44]. Vitamin D production in mothers, which depends largely on ultraviolet exposure and follows a seasonal distribution [45], directly influences fetal vitamin D levels. In utero vitamin D deficiency is known to affect the developing immune system, and genes associated with RA have been found to be significantly enriched for vitamin D receptor binding sites [46, 47]. Further, vitamin D3 has been shown to mitigate strong T-helper (Th) 1 responses while enhancing Th2 cell development [48], with gestational vitamin D3 deficiency leading to persistent alterations in the immune system into adulthood [46]. Consequently, excessive sunlight exposure can also contribute to the risk of RA, as high levels of ultraviolet radiation may impair aspects of cell-mediated immunity [49, 50]. In addition, participants in coastal regions may have long-term seafood consumption, providing abundant vitamin D supplementation. This may be a confounding factor, which could obscure the true relationship between sunlight exposure and the risk of RA. Overall, these findings underscore the complex and dual role of sunlight exposure in RA pathogenesis, where insufficient and excessive exposure may adversely affect immune regulation.

The strength of our study is that natural environmental data at birth is objective, thereby minimizing potential biases. This study’s large-scale population sample enhances the representativeness and statistical power of the results, while providing a comprehensive exploration of the impact of natural environmental factors on RA in the Chinese population. Inevitably, there are some limitations in our study. First, there is potential residual confounding from unaccounted population migration patterns as geographical relocation may modify environmental exposures during critical developmental periods. Although whole-genome sequencing data from the CKB cohort suggested that only 5.7% of participants had non-local ancestry and the actual immigration rates may be even lower [51], this factor was not systematically adjusted for in our analysis. Second, the retrospective design may introduce recall bias. Third, the study has limited generalizability due to its exclusive focus on the Chinese population, which restricts the extrapolation of findings to other ethnic groups with distinct genetic backgrounds and environmental contexts. Finally, the lack of data on relevant dietary factors (e.g., vitamin D intake) may impact the results.

## Conclusion

Although no association was found between birth month and the risk of RA, our study revealed associations between natural environmental factors like seasonality, temperature, latitude, and sunlight exposure with the risk of RA. Specifically, being born in Spring and Summer, as well as early life exposure to low latitudes and extreme sunlight environments, increases the risk of RA, which points to complex natural environmental influences on disease pathogenesis. Our results also emphasized the impact of early-life environment on the onset of RA. These findings underscore the complex influence of natural environments on disease pathogenesis and highlight the role of early-life environment on the onset of RA.

## Electronic supplementary material

Below is the link to the electronic supplementary material.

Supplementary Material 1

## Data Availability

The datasets supporting the conclusions of this study is available on the study websites (https://www.ckbiobank.org, https://www.cma.gov.cn/), along with information on access policies and application procedures. Further inquiries can be directed to the corresponding author.

## Abbreviations

RA: Rheumatoid arthritis
CKB: China Kadoorie Biobank
CDC: Chinese Centre for Disease Control and Prevention
OR: Odds ratio
CI: Confidence intervals
RCS: Restricted cubic splines
HR: Hazard ratio
