# Supplementary material for: Natural environmental factors at birth on risk for rheumatoid arthritis: the impact of season, temperature, latitude, and sunlight exposure
Source: BMC Public Health. 2025 Apr 3;25:1267. doi: 10.1186/s12889-025-22448-2 (PMC11970014; doi:10.1186/s12889-025-22448-2)
Supplement: Supplementary file 1 — Supplementary Material 1 [file 12889_2025_22448_MOESM1_ESM.docx]

**Supplementary**


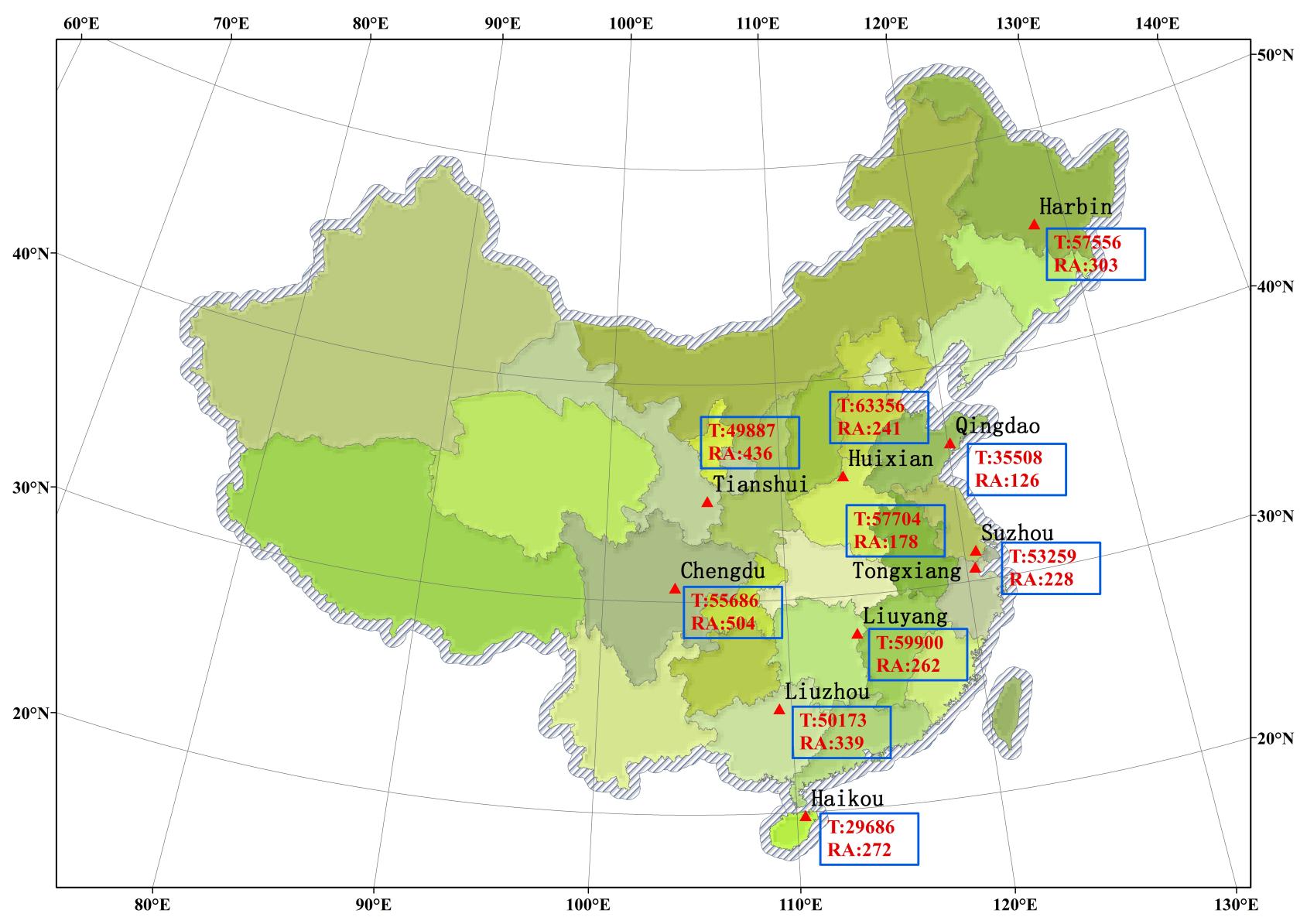


**Supplementary Fig. 1** **Geographical locations of the 10 regions throughout mainland China.** T= total.

**Supplementary Table 1 Characteristics of included regions.**

| Region | Latitude  (°N) | Climate | Average sunshine rate (%) |
| --- | --- | --- | --- |
| Qingdao | 36 | Temperate | 61 |
| Harbin | 45 | Temperate | 60 |
| Haikou | 20 | Tropical | 55 |
| Suzhou | 31 | Subtropics | 44 |
| Liuzhou | 24 | Subtropics | 37 |
| Chengdu | 30 | Subtropics | 28 |
| Tianshui | 34 | Temperate | 48 |
| Huixian | 35 | Temperate | 46 |
| Tongxiang | 30 | Subtropics | 45 |
| Liuyang | 28 | Subtropics | 36 |

**Supplementary Table 2:** **Observed and expected RA incidence numbers for each month.**

| Month | Observed numbers  (n=2,889) | Expected numbers  (n=1,563) |
| --- | --- | --- |
| January | 214 (7.4) | 115 (7.4) |
| February | 208 (7.2) | 243 (15.5) |
| March | 245 (8.5) | 114 (7.3) |
| April | 238 (8.2) | 107 (6.8) |
| May | 230 (8.0) | 108 (6.9) |
| June | 211 (7.3) | 105 (6.7) |
| July | 225 (7.9) | 116 (7.4) |
| August | 267 (9.2) | 127 (8.1) |
| September | 234 (8.1) | 126 (8.1) |
| October | 303 (10.5) | 143 (9.1) |
| November | 260 (9.0) | 130 (8.3) |
| December | 254 (8.8) | 129 (8.3) |

Data are n (%). RA= rheumatoid arthritis.
